# Study of Vulvovaginal Atrophy and Genitourinary Syndrome of Menopause and Its Impact on the Quality of Life of Postmenopausal Women in Central India

**DOI:** 10.7759/cureus.54802

**Published:** 2024-02-24

**Authors:** Samiksha C Ulhe, Neema Acharya, Aarav Vats, Awantika singh

**Affiliations:** 1 Department of Obstetrics and Gynecology, Jawaharlal Nehru Medical College, Datta Meghe Institute of Higher Education and Research, Wardha, IND

**Keywords:** quality of life, vaginal dryness, women’s health, genitourinary syndrome, vulvovaginal atrophy, postmenopausal woman

## Abstract

Background

Urogenital health is a necessary part of health for all women, especially in the postmenopausal age group. We suspected that the increased incidence of vulvovaginal atrophy (VVA) had some or other effects on the quality of life of older women. So, we aimed to study VVA/genitourinary syndrome of menopause (GSM) and its impact on the quality of life of postmenopausal women in Central India. Despite its significant prevalence and detrimental impact on women’s health, VVA/GSM is underdiagnosed and undertreated. In view of the feminization of aging, VVA management is becoming increasingly crucial. This study contributes to postmenopausal women's understanding that keeping their urogenital and sexual longevity is a critical step toward healthy living and gender equality. Given its relationship with urogynecological conditions, this study will help to evaluate both subjectively and objectively the incidence of symptoms related to VVA and its effects on the quality of life of postmenopausal women. This will eventually help to understand the need to address this issue while making postmenopausal women health-related policies. Potential remedies to overcome the obstacles currently preventing patient-HCP interactions addressing sexual health include providing communication tools to facilitate the "uncomfortable" conversation, educating women, and providing enough training for healthcare professionals.

Methods

The current study was conducted at a rural tertiary healthcare center in Central India and is a cross-sectional study. The study population taken into consideration were all the postmenopausal women between the age group 45 and 75 years with at least one vulvovaginal symptom attending the Outpatient Department (OPD). The total study sample size was 100 women. Further study was conducted by interview method using a questionnaire by the principal investigator. Data was gathered with the help of a pretested questionnaire in the patient's language. Symptoms related to GSM were studied by the vaginal symptom Bothersomeness Scale. Further, a gynecological clinical examination for the confirmation of VVA was carried out, which included a gynecological physical examination. The Vaginal Health Index (VHI) was calculated for each female using the score scale. Assessment of the quality of life of postmenopausal females using the Day-to-Day Impact of Vaginal Aging (DIVA) Questionnaire was performed.

Results

The majority of females (34%) who presented with the symptoms were in the category of 55-60 years followed by 22% in the age group of 61-65 years. The most common symptoms experienced by females were vaginal dryness (77%) followed by vaginal discharge (74%). Our study confirmed that 79% of the total females included in the study have a VHI score of less than 15, i.e., they suffer from VVA, thus presenting our incidence at 79%.

Conclusion

According to the surveys discussed in this research, a significant portion of postmenopausal women have symptoms linked to VVA that have a negative impact on their quality of life, including their sexual relationships and self-esteem.

## Introduction

Urogenital health is an essential element of postmenopausal females and their partners' healthy aging. One in every two postmenopausal women suffers from vulvovaginal atrophy (VVA), often called the "genitourinary syndrome of menopause (GSM)" [1].

Recently, VVA has been termed GSM to underline the multitude of genital, urinary, and sexual symptoms that are related to the functional and anatomical changes of vulvovaginal tissues that occur with menopause and increasing age [2].

To assess almost all the pertinent scientific documentation in the area of postmenopausal sexual and urogenital health, a terminology unison conference was conducted in May 2013, which was sponsored by the International Society for the Study of Women's Sexual Health (ISSWSH) and the North American Menopause Society (NAMS) [1]. After a two-day deliberation, recognized specialists concluded that the illness formerly known as VVA needed a new title to be used to define it more precisely. The need for a name largely admissible in the medical and public spheres, as well as the necessity to better and enhance education, communication, research, and care of sexual and urogenital problems in postmenopausal females, led to the selection of GSM. The definition of the syndrome describes various clinical signs and symptoms (genitourinary) corresponding to one another that do not have to be all present and related to solitary identifiable etiology but occur in a specific circumstance (menopause).

The definition of GSM is "a collection of symptoms and signs associated with a reduction in estrogen and other female gonad hormones that involves alterations to the vestibule, clitoris, labia majora, labia minora, vagina, urethra, and urinary bladder" [3]. Genitourinary symptoms of burning, irritation, and dryness, dysuria, urgency, incontinence, nocturia and repeated infections of the urinary tract and sexual symptoms of pain or discomfort, lack of lubrication, and postcoital bleeding are all possible indications of the syndrome [2]. "Females might present with few or all of the symptoms and signs that must be bothersome and should not be better accounted for by another diagnosis" [2].

VVA is caused by a drop in estrogen levels in the blood, which is a classic sign of menopause. Subjective (the most bothersome symptoms technique) and objective (calculation of vaginal maturation index and vaginal pH) assessments must be performed in both clinical and research contexts. A study conducted in Europe confirmed the presence of VVA in 90% of females who attained menopause by gynecological examination [4]. Since life expectancy has grown, many women are likely to be in the postmenopausal period for at least one-third part of their life, which is a hypoestrogenic state which typically occurs between 51 and 55 years of age. Menopausal syndrome is a multidimensional condition that consists of intrapersonal and interpersonal elements that vary depending on the sociocultural context and the healthcare system modification of biological variables. VVA is just one of the numerous changes that follow menopause as a result of the aging ovaries' decreased ability to produce estrogen. Although less frequently, it could result from other hypoestrogenic conditions. Contrary to hot flushes, which typically go away with time, VVA is chronic and progressive throughout the menopause transition, and the presence and intensity of symptoms vary from mild discomfort to severe discomfort, also depending upon the age of the female, time, and type of menopause, frequency of coital activity, parity and vaginal delivery, cigarette smoking, and other medical conditions/medications. To maintain proper function and structure, the vagina and the tissues around the urogenital organs need estrogen stimulation. During reproductive life, receptors of estrogen are extensively distributed in the vagina, vulva, endopelvic fascia, muscles of the pelvic floor, urethra, and trigone of the bladder. These receptors diminish after menopause and can be reinstated by estrogen therapy. Lack of stimulation by estrogen leads to a decrease in the elasticity of mucosa by stimulating fusion and collagen fiber hyalinization and fragmentation of elastin fibers beyond.

With a decrease in hyaluronic acid and intercellular acid mucopolysaccharide, there is a reduction in the hydration of mucosa of the dermal layer. The rugae, the epithelial folds that permit distension, are lost, and the vagina shortens and narrows. The amount of vaginal transudate and other secretions decreases as the vaginal mucosa, introitus, and labia minora become pale and thin, and vascular supply is significantly reduced. As a sign of an estrogen-deprived vaginal squamous epithelium, there is a growing preeminence of basal/parabasal cells over intermediate cells and superior cells over time. The friable epithelium eventually bleeds with minor stress. With thinning of the cell layer of the vagina, there is also a paramount glycogen depletion and, therefore, of the inhabitants of lactobacilli, which causes a rise in the pH of the vagina (between 5.0 and 7.5) and a reduction of hydrogen peroxide in the vagina, which permits the growth of other bacteria which are pathogenic that includes group B streptococci, staphylococci, and coli forms. Similar structural and functional alterations take place in the vulva, pelvic floor, and urinary tract, impairing the neurovascular and neuromuscular substrates of the pelvis. Being a chronic illness during the postmenopausal phase, VVA can't recede unless it is properly managed [3].

According to a new Italian AGATA survey, 64.7% of females at one year and 84.2% at six years after menopause had manifested GSM [5]. Likely, an investigation done by Genisse also found that GSM was greater in females with over five years of menopause (74%) as compared to those who attained menopause for less than five years (62%) [6]. The European REal Women's VIews of Treatment Options for Menopausal Vaginal ChangEs (REVIVE) study including postmenopausal females showed that dryness of the vagina was the most general symptom (about 70%), followed by irritation of the vagina (32.7%), and dyspareunia (29.0%) [7].

There is a lack of comprehension of the health of vaginas and older females who avoid discussing VVA symptoms so easily as sexual health is a delicate subject [1]. VVA is still underdiagnosed and undertreated, in spite of its significant prevalence and negative influence on women's health. Given its link to urogynecological conditions, it is critical to give them the best possible care. GSM management that is done correctly can help postmenopausal women improve their quality of life [8]. Just 38% of the females that participated in a study conducted in the USA called REVEAL had a talk with a healthcare professional (HCP) regarding their sexual health in the recent year. Surprisingly, a few participants (about 41%) reported that they never discussed their sexual health with anybody in the previous year, not even their spouses or friends. Women frequently give reasons that fall into one of three categories asking why sexual health concerns have not been conversed: embarrassment, the conviction that nothing can be done, or the notion that the subject is inappropriate to bring up with an HCP. Other reasons were given like females thought that the HCP might be embarrassed, or the HCP was too busy, or they felt like someone else (like an HCP) should start the discussion.

According to a study by Cumming et al., most of the peri- and postmenopausal females have attempted to hide their dryness of vagina/discomfort from their husband/partner (about 61%) and about 42% of females had made excuses to circumvent intercourse due to their symptoms. Sixty-two percent of peri- and postmenopausal females who responded said that dryness of the vagina or discomfort had impacted their confidence [9]. In spite of the fact that these symptoms are distressing, they are typically dismissed by climacteric females as a normal consequence of their increasing age and menopausal state, discouraging them from seeking medical help. During the consultation, menopausal women are usually not comfortable discussing their intimate symptoms spontaneously, whereas they might find it comfortable to discuss joint pain, mood swings, hot flushes, changes in weight, and other preventive health topics like high blood pressure, risk of cancer, bone loss, or "bad" cholesterol. Moreover, postmenopausal women's hesitation to share symptoms related to VVA is also influenced by their lack of knowledge about potentially effective and safe therapies. The International Vagina Dialogue Survey, on the other hand, revealed that young women also concur that society's taboos around vaginal issues contribute to women's ignorance and that there is a critical requirement to dispel myths and obtain accurate information about vaginal and sexual health [3].

Atrophic vaginitis, also called VVA, is a medical challenge since it is unrevealed by females, underdiagnosed by healthcare professionals, and consequently undertreated. Around 50% of postmenopausal females have experienced vaginal discomfort caused by VVA [10]. VVA is a long-standing disorder that has a substantial effect on the quality of life and sexual health. Females are generally unaware of this, as well as the possibility of effective and safe treatments. Women with VVA are more likely to have genitourinary problems and female sexual dysfunction. Due to this, it is crucial to incorporate VVA in the menopause agenda by promoting an honest and reasonable discussion about intimacy and, if necessary, performing a gynecological pelvic examination. The vaginal "taboo" must be broken, suggesting the latest recommendations for the proper treatment of VVA in clinical setups, to enhance the well-being of older women [10].

In an examination of the baseline characteristics of females engaged in the hormone therapy trials of the Women’s Health Initiative, Gass et al. [11] concluded that around 61% of females between 50 and 59 years were active sexually, subsequently by 45% of females between 60 and 69 years and 28% of females aged 70 and 79 years. Females who reported vaginal dryness in the Study of Women's Health Across the Nation (SWAN) were more likely to simultaneously suffer pain at the time of intercourse and decreased arousal. Advanced menopausal status roughly doubles the incidence of sexual dysfunction. 

Cohort and Longitudinal Studies Enhancement Resources (CLOSER), an international survey, explored the effect of VVA on postmenopausal females and male partners revealing that about 55% of females and 61% of males avoid intimacy which was attributed to dyspareunia by a large percentage of females (55%) and males (61%) [12].

Levine et al. found that females who have attained menopause and are active sexually with sexual dysfunction, as described with the help of the questionnaire of Arizona Sexual Experiences Scale, were about fourfold more prone to have features of VVA as compared to those with no sexual dysfunction [13].

The above conclusions are complemented by another extensive study of females (Prevalence of Female Sexual Problems Associated with Distress and Determinants of Treatment Seeking; PRESIDE) that discovered the presence of troublesome sexual issues, which was calculated using the Female Sexual Distress Scale [14], spiked at 14.8% in middle-aged females (45-64 years) compared with females of age group 18-44 years (10.8%) or elderly females of age 65 or more (8.9%) [15].

In research by Cumming et al., 38% of perimenopausal females and 56% of postmenopausal females complained of painful intercourse because of vaginal dryness. About 78% in the perimenopausal category and 87% in the postmenopausal category believed that painful intercourse was to blame for their lower libido. The study by Cumming et al. even used the Brief Profile of Female Sexual Function to evaluate females in the peri- and postmenopausal stages for hypoactive sexual desire disorder (HSDD), and it came to the conclusion that those who tested positive for the disease mainly believed that vaginal dryness was the root cause of their decreased libido (90% of females) [16]. Similarly, a survey of females in Italy who had surgically induced menopause found that dryness of the vagina was remarkably more reported by females with reduced sexual interest as compared to those with no decrease in desire (63.2% versus 30.2%; P = 0.001) [17]. In SWAN, females complaining about the dryness of the vagina reported lower arousal and painful intercourse [18].

The PRESIDE study [19] discovered an association between urinary incontinence and distressing sexual problems in females. Treating vulvovaginal symptoms using local estrogen therapy (LET) may treat urinary complaints as well [20]. According to the Women's Voices in Menopause, 63% of females have never been provided any sort of medication for discomfort related to the vagina due to menopause, while about 19% had been given treatment in the past and only 17% were presently receiving the medications [21]. NAMS recommended the first-line therapy that includes vaginal moisturizers, a continuation of sexual act, and lubricants [22]. Lubricants are applied prior to and during sexual act to reduce tissue discomfort caused by friction; their effects are transitory. Contrarily, vaginal moisturizers are applied internally, have a longer duration of action, and reduce vaginal dryness and pH levels [23,24]. 

Presently, there are very few studies done in an Indian rural setup. Hence, we aim to study the incidence of VVA and GSM in postmenopausal women in rural Central India and thereby its effect on the quality of their life to provide a basis for the changes that need to be made in the lifestyles, healthcare, and the society to reduce the silent sufferings of many females.

## Materials and methods

Materials

The current study was conducted at a rural tertiary healthcare center. It is a cross-sectional study. The study population taken into consideration were all the postmenopausal women between the age group 45 and 75 years with at least one vulvovaginal symptom attending the OPD. Exclusion criteria included women less than 45 years and women greater than 75 years of age. The total study sample size was 100 women. This study was started after the approval of the protocol by the Institutional Ethics Committee. The study was conducted after obtaining the written informed consent from the patients.

Methods

To calculate the sample size, the Daniel formula was used, where Zα/2 is the significance level, i.e., 95% confidence interval = 1.96; P = prevalence of VVA in postmenopausal females of 90% (0.90) [5]; d = desire error of margin of 6% (0.06); n = 100 patients needed in the study.

Postmenopausal women (menstruation has been absent for a minimum of 12 months at the time of the visit), aged between 45 and 75 years old, presenting to the gynecology clinic in the hospital with at least one VVA symptom were selected. All the females were informed about the study's type and purpose, and after obtaining their written consent, they were registered for the study. Further study was conducted by interview method using a questionnaire by the principal investigator. Data was gathered with the help of a pretested questionnaire in the patient's language. It included general information like time since last menstruation, marital status, history of recent sexual activity, parity, comorbidities, and lifestyle. Further symptoms related to GSM were studied by the vaginal symptom Bothersomeness Scale in which Females rated symptoms like vaginal dryness (internal), soreness of the vagina, irritation or burning (internal), itching (internal), pain at the time of intercourse, pain at the time of intercourse at the time of penetration, bleeding at the time of intercourse, vaginal discharge, pain during exercise, urinary incontinence, increased urgency, frequent urination, other urinary difficulties, recurrent urinary tract infections, postcoital cystitis, and lower abdominal pain on a five-level ordered response scale where 0 indicates "not at all," 1 denotes "a little bit," 2 denotes "moderately," 3 denotes "quite a bit," and 4 indicates "extremely." Higher scores indicate more discomfort caused by symptoms. Individual item scores were added together to produce total scale scores. Further, a gynecological clinical examination for the confirmation of VVA was carried out, which included a gynecological physical examination. The Vaginal Health Index (VHI) [25] was calculated for each female using the score scale. VHI might make it possible to accurately and objectively evaluate age changes in a female’s urogenital tissue [26]. Assessment of the quality of life of postmenopausal females using the Day-to-Day Impact of Vaginal Aging (DIVA) Questionnaire [27] was performed, which was translated into the local language for better understanding.

## Results

Table 1 depicts the distribution of females according to age. A majority (34%) belong to the age group of 56-60 years, whereas only 6% were of the age group 71-75 years.

**Table 1:** Distribution of females according to age

Age group	Frequency	Percentage
45-50	11	11
51-55	14	14
56-60	34	34
61-65	22	22
66-70	13	13
71-75	6	6
Total	100	100

Among the total females that participated in the study, the majority (36%) of them had their last menstruation before 5-6 years. About 12% of them had their last menstruation 1-2 years ago, while 27% had their last menstruation 3-4 years back. About 25% of females reported they had their last menstruation in more than six years which is depicted in Table 2.

**Table 2:** Distribution of females according to time since the last menstruation

Time since last menstruation	Frequency	Percentage
1 to 2 years	12	12
3 to 4 years	27	27
5 to 6 years	36	36
More than 6 years	25	25
Total	100	100

According to Table 3, 86% of females had recent sexual activity, whereas only 14% of them had no recent sexual activity. 

**Table 3:** Distribution of females according to sexual activity

Recent sexual activity	Frequency	Percentage
Present	86	86
Absent	14	14
Total	100	100

According to Table 4, 93% of females are married, 2% are widow, and 5% of them are divorced or separated from their partners. 

**Table 4:** Distribution of females according to marital status

Marital status	Frequency	Percentage
Married	93	93
Widow	2	2
Divorced/separated	5	5
Total	100	100

The majority of the females (38%) had two children, and only 3% of them were nulliparous. About 36% of females reported having more than two children, and 23% of them bear a single child which is depicted in Table 5. 

**Table 5:** Distribution of females according to parity

Parity	Frequency	Percentage
None	3	3
1	23	23
2	38	38
More than 2	36	36
Total	100	100

According to Table 6, the majority of the females (87%) reported having comorbidities, whereas only 13% of them had no comorbidities associated. 

**Table 6:** Distribution of females according to the presence or absence of comorbidities

Co-morbidities	Frequency	Percentage
Absent	13	13
Present	87	87
Total	100	100

Table 7 shows the distribution of females according to lifestyle wherein maximum females (54%) reported consuming coffee and the least of them (7%) reported that they smoke. Alcohol consumption was reported by 17% of the females, whereas 22% of them did not smoke and consume coffee or alcohol. 

**Table 7:** Distribution of females according to lifestyles

Lifestyle	Frequency	Percentage
Alcohol consumption	17	17
Coffee consumption	54	54
Smoking	7	7
None	22	22
Total	100	100

Table 8 shows the incidence of symptoms of VVA/GSM. Vaginal dryness (77%) was reported the most. While only 54% of females had bleeding during intercourse.

**Table 8:** Incidence of symptoms of vulvovaginal atrophy/genitourinary syndrome

Vaginal dryness (external)	77%
Soreness of vagina	70%
Irritation or burning (internal)	72%
Itching (internal)	73%
Pain at the time of intercourse	67%
Pain during intercourse at penetration	66%
Bleeding during intercourse	54%
Vaginal discharge	74%
Pain during exercise	60%
Urinary incontinence	57%
Urine urgency	70%
Increased urine frequency	68%
Other urinary difficulties	61%
Recurrent urinary tract infections	55%
Postcoital cystitis	56%
Lower abdominal pain	56%

Table 9 depicts the rating of vaginal dryness by females wherein 29% of females rated vaginal dryness as quite a bit on the Bothersomeness Scale, while 23% of them were not at all bothered by it. Around 19% of females were moderately bothered, and 10% reported that vaginal dryness bothered them a little bit. Around 19% of females were extremely affected due to their vaginal dryness.

**Table 9:** Rating of vaginal dryness

Vaginal dryness (external)	Frequency	Percentage
Not at all	23	23
A little bit	10	10
Moderately	19	19
Quite a bit	29	29

Extremely	19	19
Total	100	100

A total of 24% of females were moderately bothered, and 28% of them were not at all bothered due to internal irritation or burning. About 24% were quite a bit bothered, whereas 15% of females reported irritation or burning as a little bit bothersome. Only 9% of females were extremely bothered which is depicted in Table 10. 

**Table 10:** Rating of irritation or burning (internal)

Irritation or burning (internal)	Frequency	Percentage
Not at all	28	28
A little bit	15	15
Moderately	24	24
Quite a bit	24	24
Extremely	9	9
Total	100	100

Table 11 shows the rating of itching (internal) where most (27%) of the females were not at all affected due to itching, whereas 26% reported itching as moderately bothersome. About 17% of females were a little bit bothered, whereas 19% of them were quite a bit bothered. Extremely bothered females were about 11%.

**Table 11:** Rating of itching (internal)

Itching (internal)	Frequency	Percentage
Not at all	27	27
A little bit	17	17
Moderately	26	26
Quite a bit	19	19
Extremely	11	11
Total	100	100

Table 12 depicts rating of pain during intercourse. Pain during intercourse was moderately bothersome for 23% of females, whereas 33% were not at all bothered. About 7% of females were quite a bit bothered due to pain during intercourse, and 37% of females reported being a little bit bothered. Zero percent of females reported that they were extremely bothered due to pain during intercourse.

**Table 12:** Rating of pain during intercourse

Pain during intercourse	Frequency	Percentage
Not at all	33	33
A little bit	37	37
Moderately	23	23
Quite a bit	7	7
Extremely	0	0
Total	100	100

Maximum of the females (36%) reported pain during intercourse at penetration as a little bit bothersome for them. Only 1% were extremely bothered. About 7% of females reported pain during penetration as quite a bit bothersome, whereas 34% were not at all bothered, and 22% of them were moderately bothered which is depicted in Table 13. 

**Table 13:** Rating of pain during intercourse at penetration

Pain during intercourse at penetration	Frequency	Percentage
Not at all	34	34
A little bit	36	36
Moderately	22	22
Quite a bit	7	7
Extremely	1	1
Total	100	100

Table 14 depicts the rate of bleeding during intercourse. About 32% of the females were a little bit bothered due to bleeding during intercourse, and 46% of them were not at all bothered. Only 1% of females were extremely bothered, whereas 3% reported quite a bit of bothersomeness. About 18% of females were moderately bothered due to bleeding during intercourse.

**Table 14:** Rating of bleeding during intercourse

Bleeding during intercourse	Frequency	Percentage
Not at all	46	46
A little bit	32	32
Moderately	18	18
Quite a bit	3	3
Extremely	1	1
Total	100	100

According to Table 15, 26% of females were not at all bothered due to vaginal discharge, whereas 9% of them were extremely bothered. About 16% of females were moderately bothered, and 28% were a little bit bothered. Around 21% of females reported vaginal discharge as quite a bit bothersome. 

**Table 15:** Rating of vaginal discharge

Vaginal discharge	Frequency	Percentage
Not at all	26	26
A little bit	28	28
Moderately	16	16
Quite a bit	21	21
Extremely	9	9
Total	100	100

Only 2% of females reported extreme bothersomeness due to pain during exercise, while 40% of them were not at all bothered. About 19% of females were a little bit bothered, whereas 27% were moderately bothered due to pain during exercise. Around 12% of females were quite a bit bothered according to Table 16. 

**Table 16:** Rating of pain during exercise

Pain during exercise	Frequency	Percentage
Not at all	40	40
A little bit	19	19
Moderately	27	27
Quite a bit	12	12
Extremely	2	2
Total	100	100

According to Table 17, about 8% of females were extremely bothered due to urinary incontinence, and 43% of them were not at all bothered. About 17% of females reported moderate bothersomeness, and 17% were a little bit bothered. About 15% of females were quite a bit bothered due to urinary incontinence. 

**Table 17:** Rating of urinary incontinence

Urinary incontinence	Frequency	Percentage
Not at all	43	43
A little bit	17	17
Moderately	17	17
Quite a bit	15	15
Extremely	8	8
Total	100	100

Table 18 depicts the rating of urinary frequency wherein majority of females (32%) were not at all bothered due to urinary frequency. Only 10% of females were extremely bothered, and 25% of females were a little bit bothered. About 14% of females were moderately bothered, whereas quite a bit of bothersomeness was reported by 19% of females.

**Table 18:** Rating of urinary frequency

Urinary frequency	Frequency	Percentage
Not at all	32	32
A little bit	25	25
Moderately	14	14
Quite a bit	19	19
Extremely	10	10
Total	100	100

About 16% of females reported a little bit of bothersomeness due to recurrent urinary tract infections, whereas 2% were extremely bothered and 45% were not at all bothered. About 23% were moderately bothered, and 14% were quite a bit bothered due to recurrent urinary tract infections according to Table 19. 

**Table 19:** Rating of recurrent urinary tract infections

Recurrent urinary tract infections	Frequency	Percentage
Not at all	45	45
A little bit	16	16
Moderately	23	23
Quite a bit	14	14
Extremely	2	2
Total	100	100

Table 20 shows the rating of postcoital cystitis in which majority of females (44%) were not at all bothered and only 1% of females were extremely bothered due to postcoital cystitis. About 9% of females were a little bit bothered, whereas 30% were moderately bothered. About 16% of females reported quite a bit of bothersomeness.

**Table 20:** Rating of postcoital cystitis

Postcoital cystitis	Frequency	Percentage
Not at all	44	44
A little bit	9	9
Moderately	30	30
Quite a bit	16	16
Extremely	1	1
Total	100	100

According to Table 21, only 1% of females were extremely bothered due to lower abdominal pain, whereas the majority of them (44%) were not at all bothered. About 9% of females reported a little bit of bothersomeness, while 16% reported quite a bit of bothersomeness. About 30% were moderately bothered due to lower abdominal pain. 

**Table 21:** Rating of lower abdominal pain

Lower abdominal pain	Frequency	Percentage
Not at all	44	44
A little bit	9	9
Moderately	30	30
Quite a bit	16	16
Extremely	1	1
Total	100	100

Table 22 depicts the total scale scores in which majority of females about 50% experienced mild discomfort due to the symptoms of VVA whereas 48% of them had moderate discomfort. Only 2% of females had severe levels of discomfort.

**Table 22:** Total scale scores

Category (symptoms)	Frequency	Percentage
Mild discomfort (0-20)	50	50
Moderate discomfort (21-40)	48	48
Severe discomfort (41-60)	2	2
Very severe discomfort (61-80)	0	0
Total	100	100

Table 23 depicts the frequency according to the VHI. A score of less than 15 denotes atrophy of the vagina, and majority (51%) of females in this study had a score between 11 and 15. Only 5% of females had a score in the range of 21-25. About 7% of females had scores of less than 5, whereas 21% of them had scores between 5 and 10. About 16% of females had a score in the range of 16-20.

**Table 23:** Vaginal Health Index

Score	Frequency	Percentage
Less than 5	7	7
5-10	21	21

11-15	51	51
16-20	16	16
21-25	5	5
Total	100	100

Table 24 summarizes the quality-of-life scores of females in different domains. The most affected domain was sexual functioning with a history of recent sexual activity with a mean of 2.00 ± 0.55 and comparatively less affected among all was the activities of daily living (mean 1.07 ± 0.48).

**Table 24:** Quality-of-life scores of females in different domains

Domains	N	Minimum	Maximum	Mean	Std. deviation	Median
Activities of daily living	100	0	2.40	1.07	0.48	1.00

Emotional well-being	100	0	3.00	1.35	0.66	1.50
Sexual functioning with a history of recent sexual activity	86	0.75	3.50	2.00	0.55	2.00
Sexual functioning regardless of sexual activity status	100	0.40	3.20	1.83	0.61	1.60
Self-concept and body image	100	0	3.00	1.57	0.62	1.60

## Discussion

The present study was conducted among postmenopausal females with the symptoms of VVA/GSM at a Medical College in Central India. The aim was to study the VVA/GSM and its impact on the quality of life of postmenopausal women in Central India. The symptoms of VVA/GSM were studied using the vaginal Bothersomeness Scale. Further, a gynecological clinical assessment for the confirmation of VVA was carried out, where the VHI was calculated for each female using the score scale. Quality-of-life scores was calculated using the DIVA questionnaire.

Though the quality-of-life study is an essential component for treatment and research purposes, very constricted information is available about the same in Central India. So, this research may add to the database helping the same. The majority of females (34%) who presented with the symptoms were in the category of 55-60 years followed by 22% in the age group of 61-65 years (Table 1). Since all the females considered in this study were postmenopausal, the majority of them had their last menstruation 5-6 years ago with a whopping 36% (Table 2). Among the total females, 93% were married, whereas 2% of them were widows and 5% of the females were divorced or separated from their partners (Table 3). About 86% of females had recent sexual activity, whereas only 14% of them had no recent sexual activity (Table 4). Almost 74% of the females have two or more children (Table 5). According to the data collected, 87% of the females were suffering from some or other kind of morbidity, whereas only 13% of them were free from morbidities (Table 6). About 54% of females reported having consumed caffeine at some point in their life or are continuing to do so, whereas 17% of females consumed alcohol and only 7% are recorded to smoke (Table 7).

From the data collected, most of the symptoms were observed in the majority of females. The most common symptoms experienced by females were vaginal dryness (77%) followed by vaginal discharge (74%). The least commonly experienced symptom was bleeding during intercourse (54%) and recurrent urinary tract infections (55%). Other symptoms observed were soreness of the vagina in 70% of females, irritation or burning (internal) in 72%, itching in 73%, pain during intercourse in 67% of females, pain during intercourse at penetration in 66%, pain during exercise in 60%, and lower abdominal pain in about 56% of females. Urinary symptoms were also commonly observed like urinary incontinence in 57% of females, urinary urgency in 70% of females, increased urinary frequency in 68% of females, other urinary difficulties in about 61% of females, and postcoital cystitis was also much more commonly encountered in almost 56% of females (Table 8).

Nappi et al. found in their study that 78.3% of females had vaginal dryness, and 82.6% of them had experienced pain during intercourse [28]. Angelou et al. observed vaginal dryness in about 90% of females, pain during intercourse in about 80% of females, and urinary urgency in 28% of them [29]. Ojha et al. reported vaginal dryness in 78.2% of females and irritation, itching, and urinary urgency in about 54% of females [30]. The Vaginal Health: Insights, Views, and Attitudes (VIVA) study revealed the prevalence of specific symptoms among 1578 females with vaginal discomfort as follows: dryness of the vagina, 83%; pain while having intercourse, 42%; urinary incontinence, 30%; soreness of vagina, 27%; itching, 26%; burning sensation, 14%; and pain while touching the vagina, 11%. This study covered the widest range of countries. The majority of uncomfortable women (62%) in the same survey rated the intensity of their clinical features as moderate or severe. Komi and Santoro discovered that 42% of postmenopausal females who were not taking estrogen at the time reported dryness of the vagina and pain during intercourse as being moderately or very bothersome [15].

The majority of females about 50% experienced mild discomfort due to the symptoms of VVA, whereas 48% of them had moderate discomfort. Only 2% of females had a severe level of discomfort (Table 22). Vulvovaginal discomfort was recorded for women who presented with more than one symptom. Severity was noted on the Bothersomeness Scale and was further clinically examined by the gynecologist for confirmation of VVA. Since vaginal atrophy is defined as a VHI score of less than 15, our study confirmed that 79% of the total females included in the study have a VHI score of less than 15, i.e., they suffer from VVA, thus presenting an incidence of 79% (Table 23).

A sample of Italian women seeking a standard gynecological examination was enrolled in the AGATA study, and clinical diagnoses of VVA were shown to be prevalent from one to six years after menopause, with prevalence ranging from 64.7% to 84.2% [5]. The quality of life was assessed using the DIVA questionnaire. The quality of life was divided into five domains, namely, the daily activities domain, emotional well-being domain, sexual functioning domain (shorter version), sexual functioning domain (longer version), and self-concept and body image domain. According to Ojha et al., vaginal symptoms had the least impact on daily activities (mean score: 0.6 ± 0.7) and some effect on emotional well-being (mean score: 1.0 ± 0.8) and body image (mean score: 1.2 ± 0.7). The highest effect was seen in the sexual functioning who were not sexually active (mean score: 1.8 ± 1.0) [30].

The research conducted in the Western setup had a mean score of 0.6 as far as the activity of daily living is concerned. As these women are well aware of the medical condition under discussion, thereby seeking medical care or the same in most cases. It is an interesting fact that in both the study setups the minimum score was obtained in the “activity of daily living” domain. Indian rural setup gained more scores than the Western setup in this criterion because of certain obvious reasons. When we approached females for our study, it was then only when they realized that most of the questions raised had an associated symptom as far as their lives were concerned. Most of the time, these women don’t prioritize symptoms of these conditions as they do consider them to be a part of normal aging. These lead to poor monitoring and thereby eventually seeking lesser medical aid for the management of such medical conditions.

The lack of awareness is undoubtedly a very great burden on women of the rural population in Central India. Though the difference was seen in all the domains listed, the most significant variation is seen in the domain of activity of daily living being emphasized. Mental health is an important point of discussion nowadays. It was necessary to include the domain of emotional health in the study to understand how it affects females, and the results have made us think that it is necessary to prioritize emotional health along with physical health. As far as the domain of emotional well-being is concerned, our study had a mean of 1.35, and the same Western study is 1.00 which is relatively comparable. The results of our study could be related to the fact that there is a huge stigma associated with such issues, and thus, the discussion on such topics seldom becomes important among females and their partners or HCPs. This ultimately leads to frustration and depression among females and worsens their emotional and mental health. This study was conducted in a rural setup, which adds to the severity of the issue as emotional and mental health is rarely a matter of concern for the people residing over here.

According to our study, the mean score for the domain of sexual functioning with a history of recent sexual activity is 2.00, and the mean score for sexual functioning regardless of sexual activity status is 1.83. Western studies had a mean score of 1.8 in the females who were not sexually active. Symptoms of VVA have a significant impact on the confidence of females to sexually satisfy their partners. In females with a recent history of sexual activity, it was observed that there was a noteworthy impact on their desire to have sexual intercourse or any sort of sexual activity. The most negative impact was noted on their ability to be spontaneous about sexual activity. These reasons lead to stress in relationships with their partners. Sexual pleasure is a fundamental right of all females which is greatly affected due to the symptoms of VVA.

Our study had a mean score of 1.57 for the domain of self-concept and body image, and the Western study shows a mean of 1.2 [30]. The probable reasons for this result may be attributed to the fact that symptoms of VVA create a lot of self-doubt among the females which leads to a decrement in their confidence as they feel why only they are going through this, but the reality is that many of their peers too are undergoing such issues. They feel that they have become undesirable due to a lack of open conversation on this topic with their partners. Lack of awareness of the fact that medical treatments for such issues are available and their quality of life can be improved is undoubtedly the main culprit.

This study included females in rural Central India, where most of them were not economically stable, and hence, they seek less medical treatment for their condition leading to further deterioration of their health. Females included in our study were in the age group of 45 to 75 years, which is mostly a financially dependent category in rural setups so there was more hesitation in such females to disclose their vaginal symptoms to seek medical help.

## Conclusions

According to the surveys discussed in this research, a significant portion of postmenopausal women have symptoms linked to VVA that have a negative impact on their quality of life, including their sexual relationships and self-esteem. Also, we need to address some strengths of our study. This study included a face-to-face session to complete the questionnaires using the objective physical examination, which is a cornerstone in the detection of VVA, in contrast to other surveys previously published that were conducted over the phone or online. Face-to-face interactions have few benefits over other data collection techniques, including a better level of self-disclosure, the ability to record nonverbal cues that may alter the quality of an answer, and the ability to keep participants' attention on the task at hand. This study used the standardized questionnaire for assessing the quality of life of postmenopausal females which aids in more precise analysis.

The limitation of this study was the duration of the study, as this research was conducted over two months. We could only report the incidence of VVA based on the number of patients that attended the hospital within the given period. So, there is a possibility that the incidence in rural Central India may vary. The sample size of the study was 100 females, thereby limiting the spread of awareness among a large population. So arises the need for the conduction of more such studies to increase awareness and know in depth about the topic. Further research should assess regional variations in women's experiences, particularly those that pertain to their access to treatment alternatives and the healthcare system. The improved diagnosis and management of VVA depend on honest communication between patients and doctors regarding the midlife vulvovaginal changes and effective, well-tolerated management options for menopause-related vulvovaginal discomfort.

## Appendices

**Table 25:** Vaginal Health Index (VHI) A score of less than15 according to the vaginal health index correlates to vaginal atrophy.

Score	1	2	3	4	5
Elasticity	None	Poor	Fair	Good	Excellent
Volume of fluid (pooling of secretion)	None	Scanty amount, the vault is not entirely covered	Superficial amount, vault entirely covered	A moderate amount of dryness (small areas of dryness on cotton tip applicator)	Normal amount (fully saturates on cotton tip applicator)
pH	≥6.1	5.6-6.0	5.1-5.5	4.7-5.0	≤4.6
Epithelial integrity	Petechiae noted before contact	Bleeds with light contact	Bleeds with scraping	Not friable-thin epithelium	Normal
Moisture (coating)	None, surface inflamed	None, surface not inflamed	Minimal	Moderate	Normal

DIVA questionnare

We are interested in understanding the impact of vaginal symptoms such as vaginal dryness, soreness, irritation, and itching on your day-to-day life. For each question below, please check the answer that best describes how your activities, relationships, and feelings have been affected by any of these symptoms during the past four weeks.

PART A. During the past four weeks, how much have vaginal symptoms such as dryness, soreness, irritation, or itching made it uncomfortable or interfered with your ability to:

1. Walk at your usual speed?

□0                      □1             □2                  □3                             □4

Not at all       A little bit      Moderately      Quite a bit             Extremely

2. Wear the clothing or underwear you want?

□0                      □1             □2                  □3                             □4

Not at all       A little bit      Moderately      Quite a bit             Extremely

3. Use the toilet or wipe yourself after using the toilet?

□0                      □1             □2                  □3                             □4

Not at all       A little bit      Moderately      Quite a bit             Extremely

4. Sit for more than an hour?

□0                      □1             □2                  □3                             □4

Not at all       A little bit      Moderately      Quite a bit             Extremely

5. Get a good night’s sleep?

□0                      □1             □2                  □3                             □4

Not at all       A little bit      Moderately      Quite a bit             Extremely

PART B. During the past four weeks, how often have vaginal symptoms such as dryness, soreness, irritation, or itching caused you to feel:

6. Depressed or down?

□0                      □1               □2                   □3                       □4

Never             Rarely            Sometimes     Fairly often           Very often

7. Embarrassed?

□0                      □1               □2                   □3                       □4

Never             Rarely            Sometimes     Fairly often           Very often

8. Frustrated or resentful?

□0                      □1               □2                   □3                       □4

Never             Rarely            Sometimes     Fairly often           Very often

9. Bad about yourself?

□0                      □1               □2                   □3                       □4

Never             Rarely            Sometimes     Fairly often           Very often

PART C. The following questions ask about the impact of your symptoms on vaginal sexual intercourse as well as other types of sexual activity such as self-stimulation or masturbation. During the past four weeks, have vaginal symptoms such as dryness, soreness, irritation, or itching affected:

10. Your desire or interest in having sexual intercourse or other types of sexual activity (including self-stimulation or masturbation)?

□0                      □1             □2                  □3                             □4

Not at all       A little bit      Moderately      Quite a bit             Extremely

11. How frequently you had sexual intercourse or other types of sexual activity (including self-stimulation or masturbation)?

□0                      □1             □2                  □3                             □4

Not at all       A little bit      Moderately      Quite a bit             Extremely

12. Your ability to become aroused during sexual activity (including self-stimulation or masturbation)?

□0                      □1             □2                  □3                             □4

Not at all       A little bit      Moderately      Quite a bit             Extremely

Not applicable: I have not had sexual activity of any kind recently

13. Your ability to be spontaneous about sexual activity (including self-stimulation and masturbation)?

□0                      □1             □2                  □3                             □4

Not at all       A little bit      Moderately      Quite a bit             Extremely

Not applicable: I have not had sexual activity of any kind recently

14. The amount of pleasure you experienced during sexual activity (including self-stimulation or masturbation)?

□0                      □1             □2                  □3                             □4

Not at all       A little bit      Moderately      Quite a bit             Extremely

Not applicable: I have not had sexual activity of any kind recently

15. Your desire or interest in being in a sexual relationship?

□0                      □1             □2                  □3                             □4

Not at all       A little bit      Moderately      Quite a bit             Extremely

16 Your confidence that you could sexually satisfy a partner?

□0                      □1             □2                  □3                             □4

Not at all       A little bit      Moderately      Quite a bit             Extremely

17. Your overall satisfaction with your sex life?

□0                      □1             □2                  □3                             □4

Not at all       A little bit      Moderately      Quite a bit             Extremely

PART D. The following statements describe ways in which your vaginal symptoms may have affected your feelings about yourself and your body. Please indicate how true each of the following statements has been for you during the past four weeks.

18. My vaginal symptoms make me feel like I’m getting old.

□0                      □1                      □2                           □3                        □4

Not at all true   A little true   Somewhat true       Mostly true           Definitely true

19. I feel undesirable because of my vaginal symptoms.

□0                      □1                      □2                           □3                        □4

Not at all true   A little true   Somewhat true       Mostly true           Definitely true

20. When I think about my vaginal symptoms, I feel like I have lost something.

□0                      □1                      □2                           □3                        □4

Not at all true   A little true   Somewhat true       Mostly true           Definitely true

21. My vaginal symptoms make me feel like my body is deteriorating.

□0                      □1                      □2                           □3                        □4

Not at all true   A little true   Somewhat true       Mostly true           Definitely true

22. I feel less sexy because of my vaginal symptoms.

□0                      □1                      □2                           □3                        □4

Not at all true   A little true   Somewhat true       Mostly true           Definitely true

Thank you!
